# Modified Nonlinear Hysteresis Approach for a Tactile Sensor

**DOI:** 10.3390/s23167293

**Published:** 2023-08-21

**Authors:** Gasak Abdul-Hussain, William Holderbaum, Theodoros Theodoridis, Guowu Wei

**Affiliations:** School of Science, Engineering and Environment, University of Salford, Salford M5 4WT, UK; w.holderbaum@salford.ac.uk (W.H.); t.theodoridis@salford.ac.uk (T.T.); g.wei@salford.ac.uk (G.W.)

**Keywords:** conductive fabric, hysteresis, backpropagation neural network, tactile sensor

## Abstract

Soft tactile sensors based on piezoresistive materials have large-area sensing applications. However, their accuracy is often affected by hysteresis which poses a significant challenge during operation. This paper introduces a novel approach that employs a backpropagation (BP) neural network to address the hysteresis nonlinearity in conductive fiber-based tactile sensors. To assess the effectiveness of the proposed method, four sensor units were designed. These sensor units underwent force sequences to collect corresponding output resistance. A backpropagation network was trained using these sequences, thereby correcting the resistance values. The training process exhibited excellent convergence, effectively adjusting the network’s parameters to minimize the error between predicted and actual resistance values. As a result, the trained BP network accurately predicted the output resistances. Several validation experiments were conducted to highlight the primary contribution of this research. The proposed method reduced the maximum hysteresis error from 24.2% of the sensor’s full-scale output to 13.5%. This improvement established the approach as a promising solution for enhancing the accuracy of soft tactile sensors based on piezoresistive materials. By effectively mitigating hysteresis nonlinearity, the capabilities of soft tactile sensors in various applications can be enhanced. These sensors become more reliable and more efficient tools for the measurement and control of force, particularly in the fields of soft robotics and wearable technology. Consequently, their widespread applications extend to robotics, medical devices, consumer electronics, and gaming. Though the complete elimination of hysteresis in tactile sensors may not be feasible, the proposed method effectively modifies the hysteresis nonlinearity, leading to improved sensor output accuracy.

## 1. Introduction

Tactile sensors have gained immense popularity in robotics and automation systems in applications such as artificial skins, as they provide pressure maps through a force sensor array. However, hysteresis can introduce nonlinearity and measurement errors in tactile sensors and actuator systems, thereby limiting the performance of tactile sensors. To address this challenge, several novel approaches have recently been proposed. One approach focuses on adapting the external loop of the sensor’s control system to compensate for hysteresis [1]. By estimating the hysteresis error based on the difference between the desired and the measured forces, this modification improves the accuracy and performance of robotic systems by relying on tactile sensors. In the field of piezoelectric actuators, a method for modeling hysteresis patterns using a recurrent neural network (RNN) was introduced [2]. This network captures the complex nonlinear relationship between the input voltage and output displacement, enabling hysteresis compensation and thus enhancing positioning system accuracy. Furthermore, a generalized Prandtl–Ishlinskii model was employed to compensate for hysteresis in tactile sensors [3]. By training the model with experimental data, real-time sensor output can be determined, effectively improving force measurement accuracy. Additionally, innovative designs have been developed to minimize hysteresis in tactile sensors, such as a novel soft tactile electronic skin that reduces mechanical and electrical memory effects contributing to hysteresis [4].

Xiaoliang Chen et al. introduced a novel vibration sensor designed to overcome the limitations of conventional Micro-Electro-Mechanical System (MEMS) vibration sensors [5]. This sensor utilized a suspended sensing membrane with a channel-crack design, enhancing sensitivity and stability, demonstrating a wide vibration response range from 0.1 to 20,000 Hz and a dynamic range of 0.24–100 m/s^2^. The sensor’s flexibility allowed for direct attachment to various surfaces, making it suitable for diverse applications such as energy harvesting and human-machine interaction.

Similarly, a method utilizing a Gaussian process with sensory Markov properties was proposed to counteract hysteresis in tactile sensors [6]. By incorporating current and past sensory data, the current approach models the hysteresis behavior and estimates the sensor’s output, improving force measurement accuracy in robotic systems. In the field of smart-material systems, an inverse feedforward controller utilizing a Preisach model was introduced to control hysteresis nonlinearities [7]. This method estimates the hysteresis behavior and generates an inverse feedforward control signal to compensate for the nonlinearities, enhancing the performance and accuracy of smart-material systems. Furthermore, a Generalized Prandtl–Ishlinskii Model (GPIM) inversion technique was proposed to compensate for hysteresis in micropositioning control systems [8]. By modelling the hysteresis behavior using a GPIM and generating an inverse GPIM-based compensation signal, the accuracy and performance of the micro-positioning control system can be improved. In the field of piezoelectric actuators, a modified Prandtl–Ishlinskii (PI) model was introduced to accurately capture the asymmetric hysteresis behavior [9]. Several studies have explored hysteresis compensation in soft sensors. For instance, a modified GPIM has been developed for modeling the asymmetric hysteresis nonlinearity of pneumatic artificial muscles (PAMs) [10]. Inspired by a previous study [11], the current study aimed to explore a novel and scalable method for fabricating superhydrophobic hierarchical structures for water energy harvesting using Triboelectric Nanogenerators (TENGs). Their work demonstrated significant success in addressing hysteresis nonlinearity in conductive fiber-based tactile sensors through the use of a Backpropagation Neural Network (BPNN) method. This study sought to investigate the potential of applying similar techniques to water energy harvesting in TENGs, contributing to advancements in water energy utilization technologies, building upon previous study findings.

In another study, a novel hysteresis model for piezoelectric actuators was introduced, incorporating memory effects [12]. 

Fatima et al. [13] presented a novel, low-cost pressure sensor matrix aimed at monitoring stroke patients during physiotherapy sessions. The matrix comprises a 4 × 4 flexible pressure sensor array, enabling accurate measurement of patients’ performance and progress in physical exercises. To achieve precise positioning accuracy, an artificial intelligence (AI)-based algorithm was developed and tested, showing superior results, with a mean error of 0.103 cm, compared to conventional mathematical analyses (mean error of 0.704 cm).

The proposed pressure sensor matrix offers several advantageous features, such as cost-effectiveness, ease of fabrication, high sensitivity, robustness, and flexibility, in addition to the use of paper as a structural material, which further enhances these benefits. Despite variations in individual sensor responses, the matrix demonstrates effective recognition of activity and enables assessment of stroke patients’ recovery during exercises.

Several approaches have been employed to mitigate hysteresis in soft sensors, such as recurrent neural networks (RNNs) [14]. An improved method involving a radial basis function neural network has been proposed for hysteresis modelling and correction in soft sensors [15]. Additionally, an adaptive approach employing a fuzzy neural network has been applied for hysteresis modelling and compensation in soft sensors [16]. Furthermore, a hybrid neural network has been developed as an improved method for hysteresis compensation in soft sensors [17].

In the context of conductive fiber-based tactile sensors, hysteresis nonlinearity can negatively impact precision. To address this issue, the present study introduces a successful BPNN that effectively mitigates hysteresis nonlinearity, thereby enhancing sensor accuracy. However, to gain a better understanding of measurement techniques, exploring alternative methods to tackle hysteresis and drift in tactile sensors is crucial. Capacitive sensing, piezoelectric sensing, optical sensing, resonant sensing, Hall effect sensing, and strain gauge compensation are among the alternatives discussed in this literature review. Each technique offers unique advantages, such as low hysteresis, fast response times, accuracy, and stability. By examining these alternatives, this review aims to provide valuable insights into the diverse approaches in the tactile sensor field. Exploring these alternatives and potential combinations could lead to advancements in achieving even higher accuracy and robustness in tactile sensing applications [18].

In a dedicated and focused analysis [19], an in-depth exploration was conducted into the design principles and considerations of silicon piezoresistive pressure sensors. This comprehensive examination underscored the progress made in design methodologies and fabrication processes, while critically evaluating diverse design elements that wield a significant influence over accuracy and overall performance. The study delved into modelling techniques, encompassing analytical formulations and finite element method (FEM) scrutiny, as avenues for estimating pivotal parameters and addressing intricate mechanical, electrical, and thermal facets. Specific design strategies, exemplified by meander-shaped piezoresistors and optimized diaphragm configurations, were discussed in detail in relation to amplifying the sensor’s operational capabilities. The analysis also confronted hurdles associated with temperature sensitivity and doping concentration, thereby offering invaluable insights to developers of pressure sensors and furnishing them with a roadmap for prosperous integration across a spectrum of applications.

Significant progress has been made in compensating and modeling hysteresis in soft sensors based on piezoresistive materials. However, more practical and effective methods are needed to correct hysteresis nonlinearity. To address this issue, a novel approach utilizing a Backpropagation Neural Network (BPNN) is proposed in the current study. 

## 2. Materials and Methods

To assess the effectiveness of the proposed method, four sensor units with different layer configurations (1, 3, 6, and 12) were designed. The paper introduces a hysteresis compensation technique using the BPNN to enhance the accuracy of soft sensors by modifying hysteresis nonlinearity, thereby overcoming the limitations posed by hysteresis. BPNN was trained using collected force sequences and corresponding corrected resistances, leading to a significant reduction in the maximum error caused by hysteresis, as demonstrated through experimental validation.

For training the BPNN, input–output data pairs from experimental tests were employed to modify the hysteresis nonlinearity in soft sensors. This approach aimed to enhance the accuracy of soft sensors and overcome the limitations posed by hysteresis. The training process demonstrated favorable convergence, achieving a high level of accuracy.

The effectiveness of this approach was validated through experiments, showing a significant reduction in the maximum error caused by hysteresis. Specifically, the maximum error was reduced from 24.2% to 13.5% of the sensor’s full-scale output, as shown in Table 1.

## 3. Modeling the Hysteresis Nonlinearity in Conductive Fiber-Based Tactile Sensors Using BPNNs

Soft tactile sensors based on piezoresistive materials have drawn significant attention in recent years on account of their wide-ranging applications in robotics, medical devices, consumer electronics, and gaming. However, the accuracy of these sensors is often hindered by hysteresis, a nonlinear phenomenon wherein a sensor’s output is influenced by its current input and previous history. Hysteresis can introduce notable measurement errors and compromise the reliability of the sensor.

Several methods have been proposed to address hysteresis and modify the hysteresis nonlinearity of piezoresistive sensors, including curve-fitting models and neural network approaches [20,21,22,23]. Though hysteresis approximation using BPNN has already been employed, the novelty of the proposed method lies in its specific application to a conductive fiber-based tactile sensor. Furthermore, BPNN was employed to modify the hysteresis nonlinearity in this particular sensor type, which has not been explored in previous studies within the context of conductive fiber-based tactile sensors.

Notably, the novelty of research is not solely determined by the individual components or techniques utilized. Instead, it often resides in combining or applying these components in a new or unique context. In this instance, although using BPNN for hysteresis approximation is not groundbreaking, its application to conductive fiber-based tactile sensors represents a fresh approach within the specialized domain of tactile sensing.

In addressing the hysteresis phenomenon within our tactile sensor system, the Back Propagation Neural Network (BPNN) was selected as our model of choice. We recognized the existence of alternatives such as Convolutional Neural Networks (CNN), known for their adeptness in handling complex spatial relationships. However, our emphasis on balancing accuracy and simplicity led us to favor the BPNN.

While CNNs are proficient in capturing intricate patterns, their layered architecture and focus on spatial hierarchies can introduce excessive complexity for our specific application. Our goal was to find a solution that effectively mitigates hysteresis while maintaining practicality and deployability.

The BPNN stood out due to its capacity to model nonlinear hysteresis and intricate relationships common in tactile data. Its architecture strikes a balance between accuracy and complexity, avoiding unnecessary intricacies that could hinder interpretability. The model’s stability in handling noisy data aligns with our aim for reliable predictions.

While alternatives are valuable, the BPNN aligns closely with our objective of a pragmatic and precise solution. By utilizing the BPNN to adjust the sensor’s resistance based on polynomial curve approximations, we markedly enhanced the accuracy and consistency of our sensor’s outputs. This decision effectively addressed hysteresis, resulting in an optimally performing tactile sensor system.

**Figure 1:**
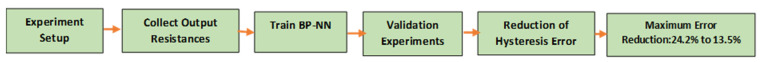
Flow Diagram of the Experiment Setup and Hysteresis Error Reduction.

Figure 1 describes the experimental setup used to design four distinct types of sensor units with varying layers, along with the process of collecting output resistances by applying force sequences. These force sequences and the corresponding corrected resistances were employed as inputs to train the BPNN, resulting in favorable convergence and high accuracy. Through validation experiments, a reduction in the maximum error caused by hysteresis was demonstrated in the proposed method, wherein the sensor’s full-scale output was reduced from 24.2% to 13.5%.

Subsequent sections delve into the theoretical background underlying hysteresis modeling, curve-fitting models, and neural networks. Additionally, we elucidate how these methods were integrated into the proposed approach, showcasing a block diagram and a specific algorithm.

### 3.1. Hysteresis Model Using a Neural Network

The hysteresis model uses a BPNN that inputs the force sequence and results in the corresponding output resistance. The neural network is trained on a set of input–output data pairs, where the input is the force sequence, and the output is the corresponding output resistance of the tactile sensor. During training, the neural network adjusts its weights to minimize the difference between predicted and actual output resistance. Once trained, the neural network can be used to predict the output resistance for any given force sequence [24,25,26].

### 3.2. Curve-Fitting Model(s)

Curve-fitting models analyze different data points to establish relationships between variables, aiding in predictions and understanding patterns, with effectiveness depending on data quality and proper model selection [27,28]. In the current study, a polynomial curve-fitting model was used to correct the hysteresis nonlinearity of the tactile sensor. The polynomial curve-fitting model is given by the following Equation (1):

(1) $$R=a_{0}+a_{1}F+a_{2}F^{2}+\ldots +a_{n}F^{n}$$

where *R* is the corrected output; *F* is the raw input, and *a*_0_, *a*_1_, and *a_n_* denote the polynomial coefficients. The coefficients are determined by minimizing the sum of the squared error between the corrected output and the actual output.

### 3.3. Neural Network

Neural networks (NNs) are computational models that learn complex patterns and relationships from data. With their interconnected layers and mathematical operations, NNs enable accurate predictions and valuable insights in various domains. In the present study, a BPNN was employed to modify the hysteresis nonlinearity of the tactile sensor [29]. The BPNN algorithm consists of two phases: forward and backward. In the forward phase, the input is propagated through the network to generate the output. In the backward phase, the error between the actual output and the desired output is propagated backwards through the network to adjust the weights of the nodes [30,31]. The algorithm can be summarized as shown in Equation (2) below:

(2) $$a_{j}^{l}=\sigma (\sum_{i}w_{ji}^{l}a_{i}^{l-1}+b_{j}^{l})$$

Here, $a_{i}^{l-1}$ represents the output of node *j* in layer *l*; $w_{ji}^{l}$ is the weight connecting node I in layer *l* −1 to node j in layer *l*; $a_{i}^{l-1}$ is the output of node I in the previous layer; $b_{j}^{l}$ is the bias of node *j* in layer *l*; and σ is the activation function.

In the backward phase, the error between the actual output and the desired output is propagated backwards through the network to adjust the weights of the nodes. The error $b_{j}^{l}$ of each node *j* in layer *l* measures how much that node contributes to the overall network error. It is defined as the partial derivative of the total error *E* for the input $z_{j}^{l}$ of node *j* in layer *l*:

(3) $$\delta_{j}^{l}=\frac{\partial E}{\partial z_{j}^{l}}$$

The weight updates are then determined based on this network error. The weights are adjusted in a direction that reduces the error by an amount proportional to the error and the previous layer’s output. This is known as the delta rule, which is represented by the following Equation (4):

(4) $$∆w_{ji}^{l}=-\eta \delta_{j}^{l}a_{i}^{l-1}$$

Here, ∆ is the learning rate, which controls the size of weight updates. The biases are updated similarly:

(5) $$∆b_{j}^{l}=-\eta \delta_{j}^{l}$$

These weight and bias updates are applied to the network after processing each input to reduce the error gradually over time. By iteratively adjusting the weights and biases, the network can learn the mapping between the input and the desired output.

The algorithm can be summarized as follows:

1. Initialize the weights and biases of the network randomly;
2. For each input in the training data:
  - Perform the forward phase to generate the output of the network;
  - Calculate the error between the actual output and the desired output;
  - Perform the backward phase to adjust the weights of the network;
  - Calculate the error between the actual output and the desired output;
3. Repeat step 2 for a specified number of epochs or until the network reaches a satisfactory level of performance (see Algorithm 1).

**Algorithm 1**: Neural Network Training**Require:** train_data: Matrix of input training data**Require:** desired_output: Matrix of corresponding desired output data**Require:** num_epochs: Number of training epochs**Ensure:** Trained neural network weights and biases 1: Initialize the network weights and biases randomly 2: **for** *epoch* = 1 to *num_epochs* **do** 3:   **for** *i* = 1 to size(training_data,1) **do** 4:     *input_data* = training_data(i,:) 5:     *output_data* = desired_output(i,:) 6:     Perform the forward phase 7:     *predicted_output* = neural_network(*input_data*) 8:     Calculate the error between the predicted output and the desired output 9:     *loss* = loss_function(*output_data*, *predicted_output*) 10:     Perform the backward phase 11:     *gradients* = backward_phase (*loss*, neural_network) 12:     Update the weights and biases of the network 13:     neural_network=update_weights(neural_network, *gradients*) 14:   **end for** 15: **end for** 16: **return** Trained neural network weights and biases

The selection of BPNN is highly strategic for effectively mitigating hysteresis nonlinearity in conductive fiber-based tactile sensors. This algorithm offers several advantages that align well with the specific challenges posed by hysteresis in this context.

First, BPNN exhibits remarkable generalization capabilities, making it exceptionally suitable for deciphering intricate relationships between input and output variables. Given the intricate and nonlinear nature of hysteresis, BPNN’s ability to capture complex mappings is crucial for achieving accurate corrections.

Moreover, the proposed tactile sensor system demands a modeling approach that strikes an optimal balance between simplicity and efficiency, while still delivering precise predictions. BPNN adeptly fulfils this requirement by offering stability and robustness during the training process. Its resilience in handling noisy or incomplete datasets assures reliable predictions even in real-world scenarios.

Further enhancing its appeal, BPNN boasts high-precision approximation abilities, a critical feature for attaining consistent and accurate tactile measurements. While more intricate neural architectures such as Convolutional Neural Networks (CNNs) or Long Short-Term Memory networks (LSTMs) exist, the inherent complexity of the tactile sensor system makes BPNN an apt and potent choice for tackling nonlinear hysteresis.

While BPNN has found application in diverse domains, its specific adaptation for mitigating hysteresis in conductive fiber-based tactile sensors might remain poorly understood. Therefore, this study significantly contributes to the literature by showcasing the efficacy of BPNN in this specialized domain. The application of BPNN leads to heightened accuracy and reliability in practical implementations, spanning domains such as soft robotics, wearables, and medical devices. This underscores the relevance and value of BPNN as a tailored solution for nonlinear hysteresis within the context of conductive fiber-based tactile sensors.

**Figure 2:**
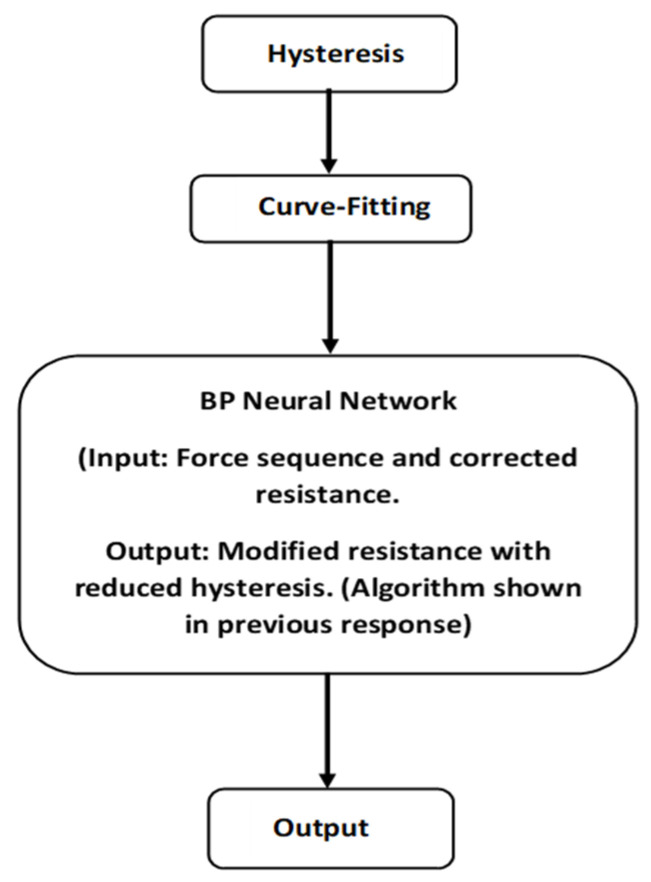
A generic flow chart showing the integration of the hysteresis model, curve-fitting model, and NN.

Figure 2 illustrates a generic block diagram showing the integration of the hysteresis model, curve-fitting model, and BPNN):

The integration of the hysteresis model, curve-fitting model, and BPNN provides a comprehensive approach for modeling a system where force is the input and resistance is the output. The hysteresis model captures the non-linear dynamics and the memory effects of the system by the hysteresis model in response to force. The curve-fitting model approximates the mathematical relationship existing between force and resistance. The BPNN learns from the curve-fitting model’s output to improve resistance predictions using backpropagation. This integrated system takes force as input, processes it through the hysteresis model, refines the output with the curve-fitting model, and further enhances it with the BPNN, thereby generating accurate predictions of resistance based on the force applied.

## 4. Design of a Soft Tactile Sensor

Fabric-based sensors were selected due to their simple design, ease of fabrication, and low cost. They are stretchable and flexible and can adhere to soft surfaces. In the following section, the fabrication and design of the tactile sensor are discussed.

### 4.1. Use of Materials

The fabric sensor employed in this study operated based on the piezoresistive effect, which induces a decrease in the electrical resistance of a piezoresistive material upon applying pressure. The sensor consists of two main components:

1. EeonTexTM knitted conductive fabric: This commercially available fabric is knitted, conductive, and stretchable; it has a thickness of 0.38 mm and a mass per unit area of 113.78 g/m2. The fabric exhibits an elongation of 40% at break and a wrap recovery of 85% after stretching. It primarily comprises 72% nylon and 28% spandex, with a proprietary conductive coating [32];
2. Silver-plated conductive thread: To establish conductive connections with measuring devices, a silver-plated conductive thread was used. MADEIRA yarn (detox 290 ± 6 HC 40) was employed for sewing [32,33].

These materials work synergistically to create a tactile sensor that detects and converts pressure changes into analogue electrical signals, typically voltage.

### 4.2. Sensor Assembly

**Figure 3:**
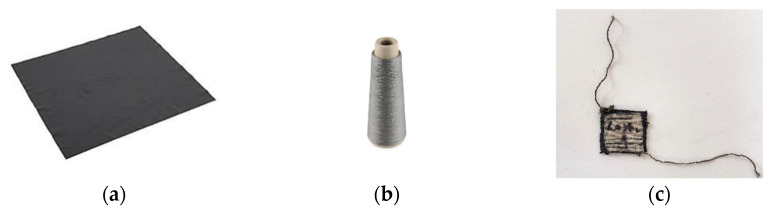
Materials used for sensor development: (**a**) conductive stretchable fabric, (**b**) silver-plated conductive thread, (**c**) designed sensor.

Assembling the sensor involved integrating the tactile sensor fabric with textiles to enable measurements of muscle activities. The silver-coated conductive thread was utilized to connect the sensor to a measurement system. This 100% polyamide fully silver-plated thread, with a linear resistance of <300 Ohm/m, was chosen for its low-resistance characteristics. In this study, the silver-plated thread was employed to optimize contact points and circuit paths [30,31]. The data for the resistance versus force relationship were collected manually and saved in an Excel file. A MATLAB program was utilized for data analysis and for generating plots to visualize the relationship between resistance and force (Figure 3).

Figure 3a displays the EeonTexTM knitted conductive fabric, which contains conductive fibers that allow electrical current to flow through it. This fabric is used in various applications, such as smart textiles, wearable technology, and sensor systems. Figure 3b displays the silver-plated conductive thread made by coating a base thread with a layer of silver. This thread is often used with conductive fabrics to create wearable electronics and others. Lastly, Figure 3c shows the completely designed sensor design that utilizes the EeonTexTM knitted conductive fabric and silver-plated thread. The objective of the specific design of the sensor is the detection of the electric resistance signals when applying force.

**Figure 4:**
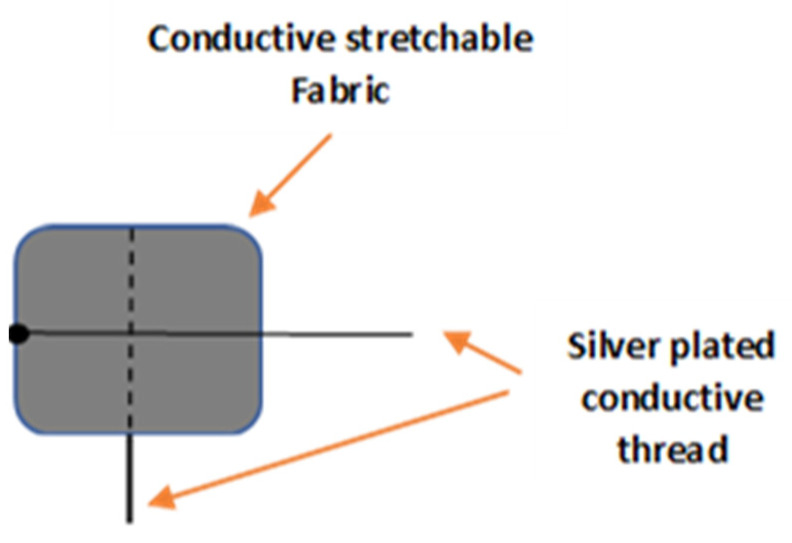
The soft tactile sensor.

To maintain the elasticity of the fabrics, the thread was sewn using a long-running loose stitch (saddle stitch). All the necessary pieces of samples were prepared with dimensions of 1 × 1 cm^2^. Figure 4 shows the overall diagram of the designed soft tactile sensor.

### 4.3. Experimental Setup

**Figure 5:**
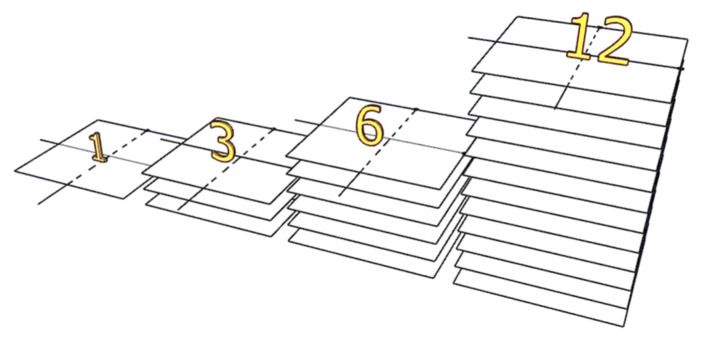
Tactile sensors with different layers.

In the current study, four tactile sensor samples (one, three, six, and twelve layers) were used, as shown in Figure 5. The sensor was designed in layers three, six, and twelve to increase the range of applying force to be measured. An increase in the number of layers implies an increase in the capacity of the tactile sensor to apply force, and it can be used in other applications, such as in the patient’s seat or bed.

**Figure 6:**
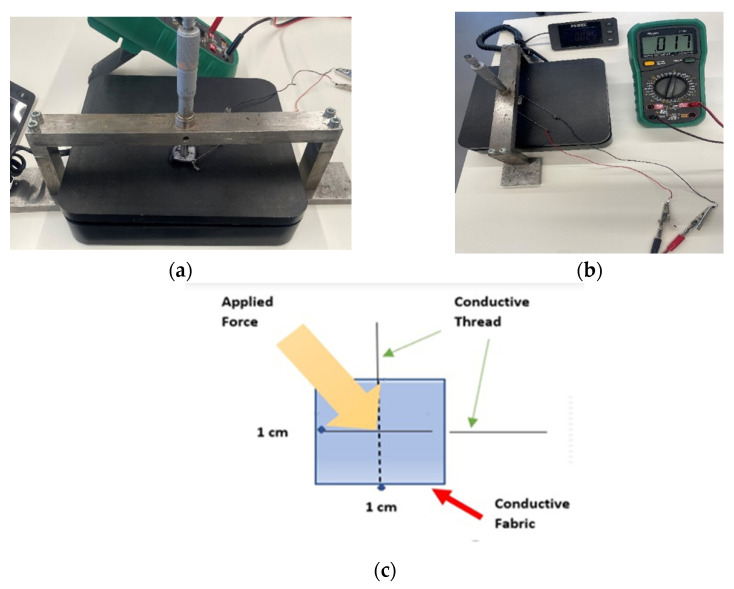
(**a**) The experimental setup, (**b**) Force being applied to the sensor, (**c**) Functional diagram.

For each sensor, force was applied using an instrument that stood over the sensor, which was installed on a digital electronic weight scale (Figure 6), after which the change in resistance was measured via a multi-meter.

Figure 6a shows the placement of a sensor on a weight scale when force is applied using a force instrument. It depicts the sensor’s location of the object being weighed or the type of force instrument being used.

Figure 6b shows a setup for measuring the resistance change in a tactile sensor. Finally, Figure 6c depicts a functional diagram (schematic) of how to apply force to a tactile sensor. This involves using a specific mechanism to exert pressure or tension on the sensor in a controlled manner.

To study the behavior of the tactile sensor output, a measurement method was used to register the sensor response to the sequences of forces that were exerted manually. Four to six consecutive loading–unloading cycles with different points of return for the same ascending curve and different starting points rising from the same descending curve were applied. Thus, the descending and ascending behaviors of the sensor were respectively characterized. The cycles were performed with an increase of 0.1 N between forces. These hysteresis curves represented the average output produced by the tactile sensor point after each cycle was repeated five times. The interval between the new force level being exerted and the resistance output was 2 s. To quantify the hysteresis exhibited by the sensor, the hysteresis error as the difference in sensor output resistance to the same applied force was determined when these forces were exerted on the ascending and descending branches of cycles. The maximum force applied by using a force instrument to the tactile sensor varied depending on the type of object used to apply the force. When using the index fingertip on a thin ring, the maximum force (F) was 59.5 ± 21.4 N, while, when using all four fingers on a straight bar, the maximum force (F) was 268.7 ± 77.2 N [32]. This variation in the F values reflected the different force ranges that the sensor was expected to encounter in real-world applications and provided essential data for understanding the sensor’s response to different levels of force.

**Figure 7:**
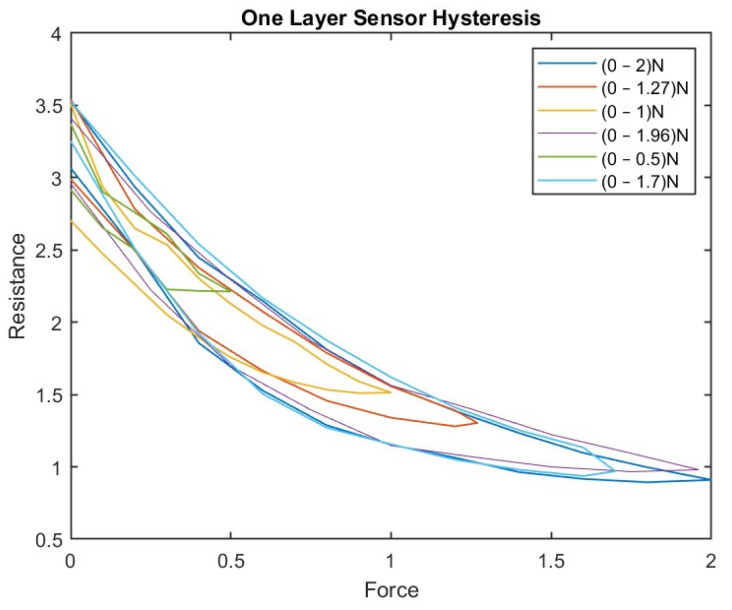
The hysteresis phenomenon in the one-layer sensor.

For the one-layer sensor, a force of 0–2 N was applied; the load was increased until the sensor output reached its saturation level. Then, the load was reduced from 2 N to 0 N, and the change in resistance was determined. Accordingly, the results were plotted as a force function. We repeated this experiment with random force ranges (0–1.27 N, 0–1 N, 0–1.96 N, 0–0.5 N, and 0–1.7 N). Figure 7 shows the hysteresis in the one-layer sensor.

The maximum and average hysteresis errors were referenced to the highest output value to obtain a percentage of the error relative to the full scale. The maximum error due to hysteresis was 24.2% of full-scale output.

Figure 7 shows the hysteresis behavior in the sensor when it was compressed to the maximum force and then released multiple times. The sensor exhibits a different number of loops, indicating variation in the sensor’s hysteresis behavior depending on the sensor’s compressed and released layer. The number of loops increases as compressions and releases increase, indicating that the force history influences the sensor’s hysteresis behavior.

For the three-layer sensor, force ranges of 0–2.8 N, 0–4 N, 0–6 N, and 0–8 N were applied, and the associated resistance was recorded over several experiments, reporting the average resistance over the individual experiments as a function of force.

**Figure 8:**
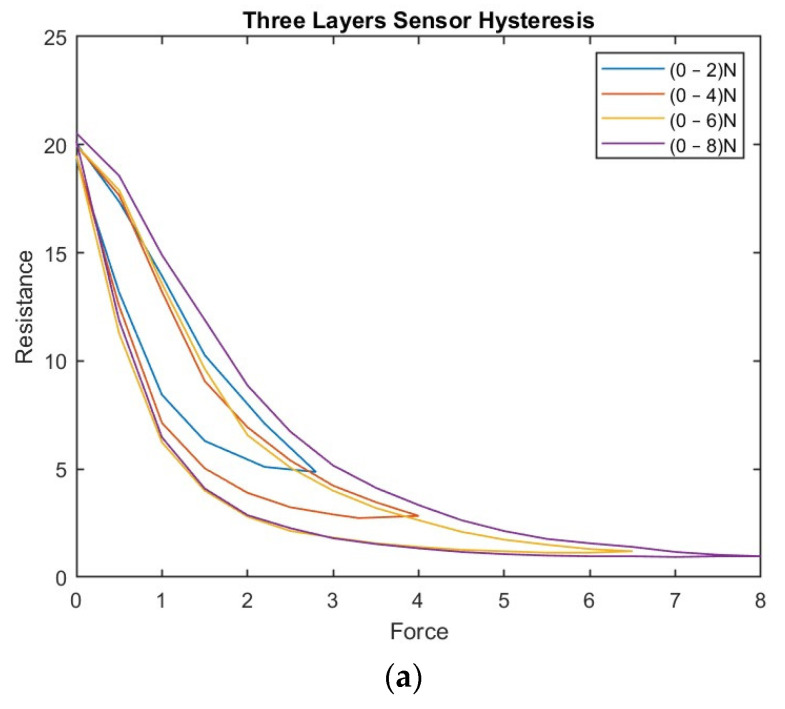

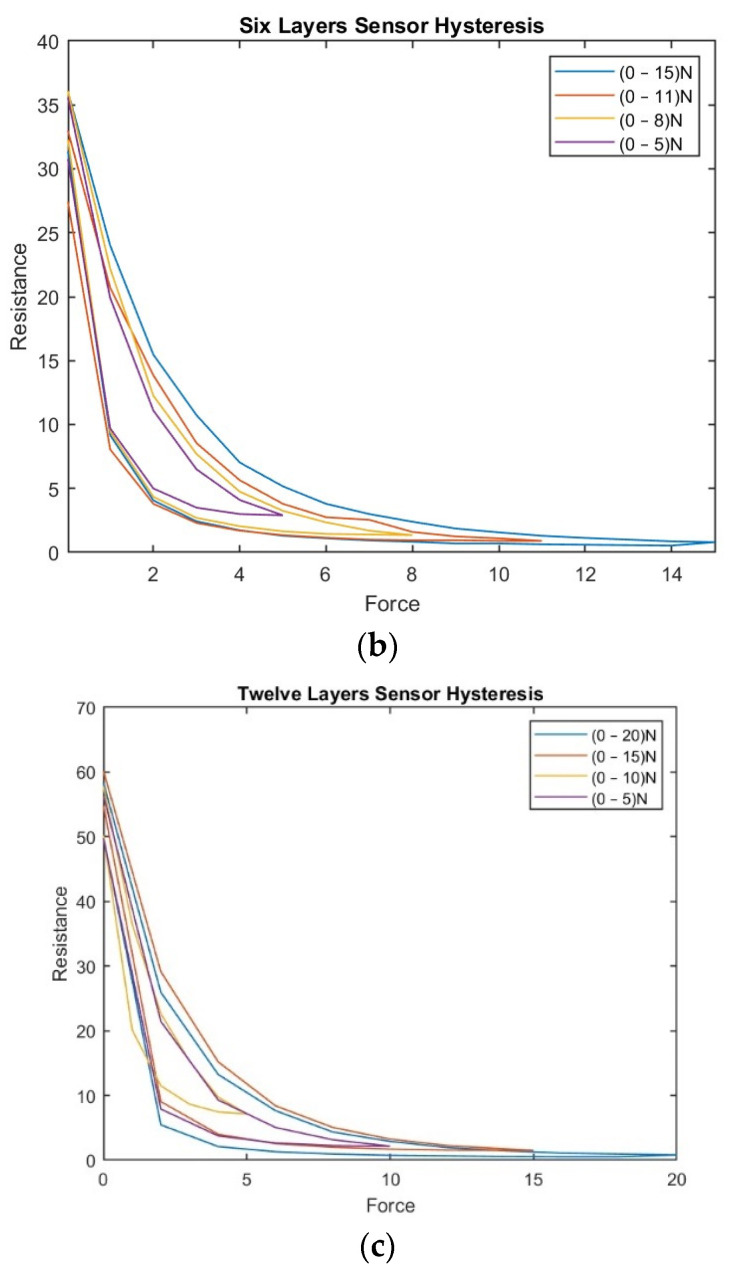
(**a**) The hysteresis phenomenon in the three-layer sensor; (**b**) The hysteresis phenomenon in the six-layer sensor; (**c**) The hysteresis phenomenon in the twelve-layer sensor.

The experiment was repeated with the 6- and 12-layer sensors, and the associated hystereses were plotted accordingly. Figure 8 shows the hystereses for the three-, six-, and twelve-layer sensors.

Both Figure 7 and Figure 8 show that the hysteresis curve of the proposed sensor exhibits a multi-loop behavior in different layers of the sensor. This implies that the output resistance of the sensor depends not only on the current force applied, but also on the force history.

Figure 8 shows the hysteresis behavior of the sensor when it is compressed and released multiple times in the same layer. The findings emphasize that the output curve of the proposed sensor is influenced by both the current force applied and the force history. This underscores the significance of considering the hysteresis during the design and interpretation of results from tactile sensors. To address this challenge, a BPNN was proposed in the current study to adjust the sensor’s resistance based on estimated values obtained from the polynomial curve. This approach enhances the accuracy and reliability of readings, enabling more precise interpretations of the sensor’s output.

## 5. Modeling Hysteresis Based on a Backpropagation Neural Network

Neural networks are a popular choice for compensating for hysteresis in tactile sensors because they can effectively learn the complex nonlinear relationships between input and output data. Tactile sensors are used to detect and measure parameters such as physical forces, pressure, and vibrations. Hysteresis, a common problem encountered in tactile sensors, can cause measurement inaccuracies. Hysteresis occurs when the sensor’s output does not return to its original state after the input has changed, causing a lag in the sensor’s response [34,35].

Neural networks can learn to compensate for this hysteresis by analyzing the sensor’s input–output data and building a model to predict the correct output based on the input. They can learn the nonlinear relationship between input and output data, allowing them to compensate for hysteresis accurately [36].

Though other methods can also be used to compensate for hysteresis in tactile sensors, such as physical calibration or mathematical modeling, these methods may be less effective in capturing the complex nonlinear relationship between input and output data. Neural networks offer a flexible and powerful solution for hysteresis compensation in tactile sensors [37].

Nonlinear hysteresis in a tactile sensor can result in degraded system performance and instability [35]. In this experiment, a BPNN was used to eliminate nonlinear hysteresis, as an Artificial Neural Network (ANN) is simple and sufficient to boost performance and reduce instability, albeit within the tactile sensor. BPNNs have a high precision approximation, robust fault tolerance, and nonlinear solid mapping capabilities [38,39,40].

BPNN was used due to its better generalization and strong nonlinear mapping abilities, which makes it a popular choice in various fields. The BPNN was trained to predict the static resistance value based on the current and historical resistance values (R_at_ and R_t−1_, respectively) and the estimated resistance value, which was used as a target [41,42,43].

The neural network used in the study consisted of an input layer that had the current resistance R_t_ at a time (t), the historical resistance R_t−1_ at a time (t − 1), and a corrected resistance (R_estimated_) as a target; the output layer was a static resistance R_Statict_ for training and two hidden layers. Each hidden layer had five neurons; the transfer function was the sigmoid function. The Levenberg–Marquardt algorithm (LMA), a popular optimization method for training neural networks, is a variation of the Gauss–Newton algorithm, which is known for fast convergence and good stability. Using the LMA algorithm to train the BPNN, the model could learn the complex relationships between the input and output variables and make accurate predictions [44].

Overall, the use of BPNN in the proposed sensor offers more accuracy and reliability, and different neural network techniques can be helpful in various applications where precision and measurement accuracy are required.

## 6. Results and Discussion

### Modified Hysteresis: Simulation and Experiments

In the current study, multiple sensor units were developed with different layers, and the output resistance was determined by applying force sequences on the sensors. These force sequences, along with the corresponding corrected resistance values, were utilized to train a BPNN. This network exhibited good convergence and demonstrated high accuracy during the training process.

A tactile sensor was used to verify the hysteresis model based on BPNN. The target part was determined by approximating a curve from the hysteresis graph, and, for each layer, the approximated curve’s line was plotted as a desired resistance.

**Figure 9:**
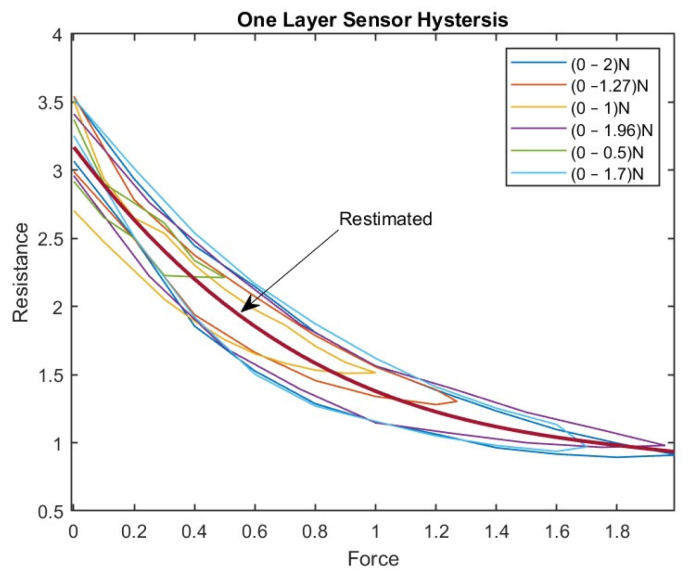
The Approximated Curve Plotted for the One-Layer Sensor.

Figure 9 shows the approximated curve plotted for a one-layer sensor. This curve represents the relationship between the sensor’s actual resistance values and the desired values (the latter are represented by the curve). The accuracy of the hysteresis model can be evaluated, and then any necessary adjustments can be made.

A third-degree polynomial curve was used to fit the hysteresis curve in a one-layer tactile sensor, which provided the best practical results. After fitting the polynomial curve, the force values were substituted into the polynomial to determine the corresponding estimated resistance values. These estimated resistance values were then used as the target input for the BPNN.

BP is a popular neural network training algorithm used to adjust the network weights and minimize the difference between the predicted and actual values [45]. In this case, the target input was the estimated resistance value obtained from the polynomial regression, and the neural network was trained to predict this value based on the input force values.

The proposed model captured the nonlinear relationship between the force and resistance values by combining the polynomial regression and neural network techniques and making accurate predictions. This approach can be helpful in various applications where complex relationships between variables are challenging for models which employ traditional techniques.

Fitting a polynomial curve to hysteresis data is a common approach, though it has limitations when the relationship is complex. In such cases, neural networks excel by capturing nonlinear patterns, extracting hidden features, generalizing to new data, and handling diverse inputs. Their flexibility and ability to model complex relationships make neural networks a preferred choice when traditional methods struggle in machine learning [28].

Once a polynomial curve is fitted to the hysteresis data, the estimated values can be used as inputs for a neural network. Neural networks are powerful machine learning models that can learn complex patterns and relationships in data. By using the estimated values from the polynomial curve as inputs for the neural network, the variables can make better predictions:

(6) $$R_{estimated}=-0.2366F^{3}+1.3822F^{2}-2.9373F+3.1703$$

where F represents the force applied (0–2 N) and $R_{estimated}$ is the estimated value of resistance. This equation shows that the estimated value of R relates to the force applied (F) in the current study. The range of values for F in this equation varies between 0 and 2 N; 41 samples were used to derive Equation (6).

**Figure 10:**
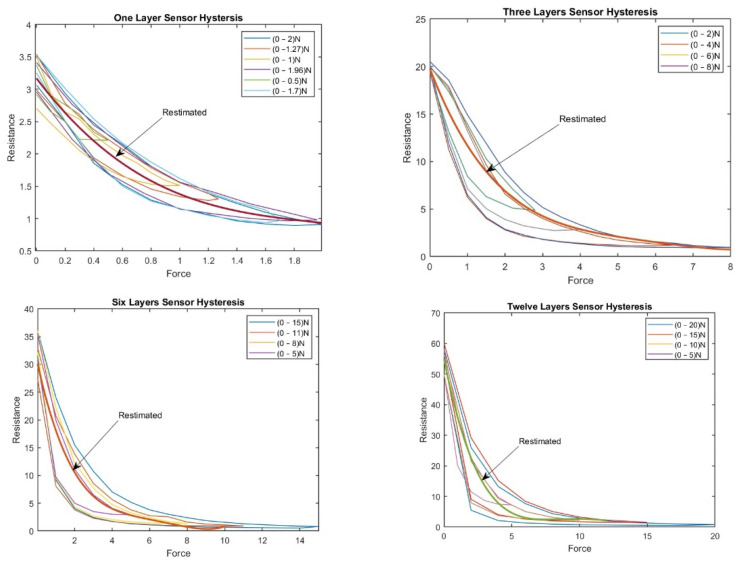
Approximated curves of all different sensors (with different numbers of layers).

The earlier tested procedure of the one-layer sensor was repeated for all four layers. Figure 10 shows the subsequent results of approximated curves of the sensors with different numbers of layers.

The corrected curve equation for all four layers of sensors can be found in the graphs shown above, and, by using the same procedure as for the one-layer sensor, the desired target in our BPNN can be found.

(7) $$R_{estimated}=0.0108F^{4}-0.259F^{3}+2.3599F^{2}-10.21F+19.765$$

The range of values for F in Equation (7) is between 0 and 8 N, and 90 samples were used to derive this equation.

(8) $$R_{estimated}=0.0125F^{4}-0.3329F^{3}+3.3176F^{2}-15.345F+30.41$$

Values for F in Equation (8) range from 0 N to 15 N. Overall, 142 samples were used to derive this equation.

(9) $$R_{estimated}=0.0056F^{4}-0.2296F^{3}+3.431F^{2}-22.182F+54.944$$

The range of values for F in Equation (9) is between 0 and 20 N, and 240 samples have been used to derive this equation. The three equations (Equations (2)–(4)) represent the estimated curve equation for the three-, six-, and twelve-layer sensors.

The equations provide an estimate of the value of R (representing a dependent variable) based on the force (F) applied, an independent variable. Each equation exhibits different characteristics in terms of the power of F and the sign of the coefficients. This helps us determine the relationship between F and R.

The power of F in the equations captures complex and nonlinear relationships between F and R, accommodating different curves and intricate patterns. By considering powers of F ranging from 2 to 4, these equations can capture the nonlinearity and complexity present in the relationship between F and R. Positive coefficients indicate a direct relationship, while negative coefficients suggest an inverse relationship. These coefficients are determined through regression analysis and depend on the dataset and the statistical method employed.

## 7. The Sensitivity of the System

Uncertainty analysis is used to quantify the uncertainty in the output of a mathematical model by examining the uncertainty in its input [41]. It aims to understand the extent of uncertainty in the output attributed to each input variable. The objective of the analysis is to conduct a sensitivity analysis using Monte Carlo simulation and understand the relationship between the force variable (F) and the estimated values of (R). This analysis is crucial for understanding the behavior of the models and evaluating the impact of force on the estimated values. This allows us to identify the most critical source of uncertainty and focus on reducing it [31,46].

Monte Carlo simulation was employed as a powerful tool to assess the sensitivity of the estimated values to different levels of applied force. The value of F was systematically varied within specified ranges to conduct the sensitivity analysis. The corresponding changes in the estimated (R) values were observed and analyzed.

The analysis revealed important findings regarding the relationship between the force variable and the estimated values of R. It provided valuable insights into how changes in force affect the estimated resistance values. By systematically varying the force variable, we observed how the estimated values of R responded, enabling a comprehensive assessment of the model’s behavior.

**Figure 11:**
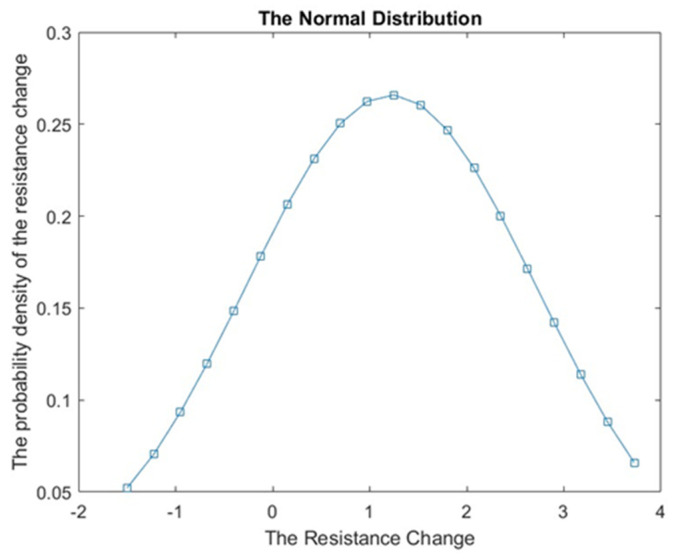
The normal distribution graph of the one-layer sensor model.

Figure 11 presents a normal distribution graph, which plays a crucial role in data analysis and prediction. The graph shows the distribution of the resistance variable within a population. The curve shown in Figure 11 indicates that the majority of values cluster around the mean, representing the center or average value. As we move further away from the mean in both directions, the number of values gradually decreases.

In the present specific case, the estimated mean distribution for the R-value was found to be 1.1125. This value serves as a measure of central tendency, indicating the average resistance value in the population. Additionally, the standard deviation of 1.513 indicates the spread of or variability in resistance values from the mean.

By analyzing the normal distribution graph and considering the mean and standard deviation, we gain valuable insights into the overall pattern and variability in resistance values.

## 8. Analysis and Discussion

The validation for the tactile sensor was performed by applying force, removing it from the sensors, and measuring the corresponding changes in resistance. The observed resistance changes were then compared to the theoretically expected resistance changes. This validation process specifically focused on evaluating the performance and accuracy of the tactile sensor itself.

**Figure 12:**
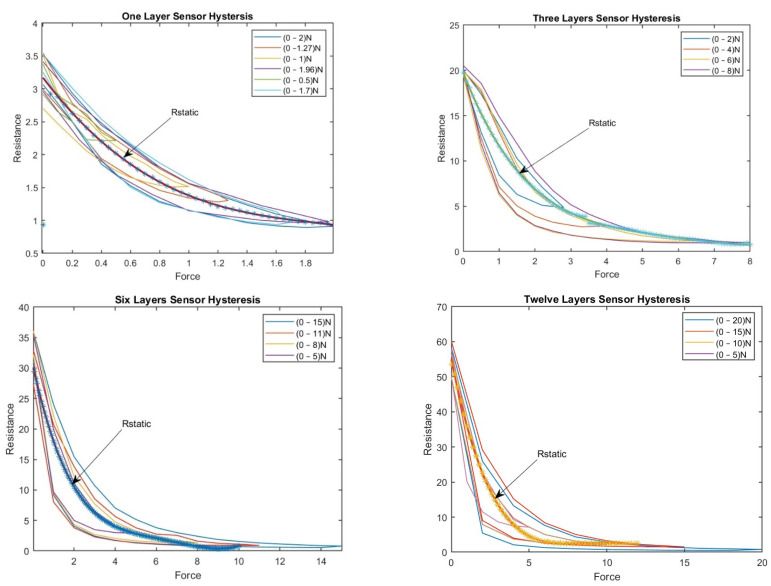
Graphs showing the validation of the system of sensors with different numbers of layers.

Figure 12 shows the validation of the system by using the same force data set as was used initially to train in the NN model for the one-layer and the other sensors.

As shown in Figure 12, the tactile sensor has been specifically designed to accurately measure changes in resistance in response to applied or removed force. Through the validation process, the tactile sensor demonstrated its capability to produce reliable and accurate results within the maximum force domain of 2 N for the one-layer sensor.

The validation results indicate that the tactile sensor consistently provides measurements that closely align with the actual values of measurements. This alignment signifies that the predicted results generated by the neural network closely match the expected values based on the training data. Notably, the output results of the BPNN, which fall perfectly on the target curve ‘R_estimated_’ after training, serve as a good indicator of the accuracy and reliability of the tactile sensor.

Furthermore, the use of the same dataset for training and validation purposes facilitates a direct comparison between the predicted and actual results. When the predicted results closely resemble the actual results, the accuracy and reliability of the tactile sensor are confirmed in measuring resistance change.

The above findings show the reliability and accuracy of the tactile sensor in measurements. It consistently provides highly dependable results and demonstrates high alignment between predicted and actual values. Accurate capture of the resistance changes, reliable performance within the specified force range, and close alignment with actual results validate the reliability and accuracy of the tactile sensor in measuring resistance changes.

**Figure 13:**
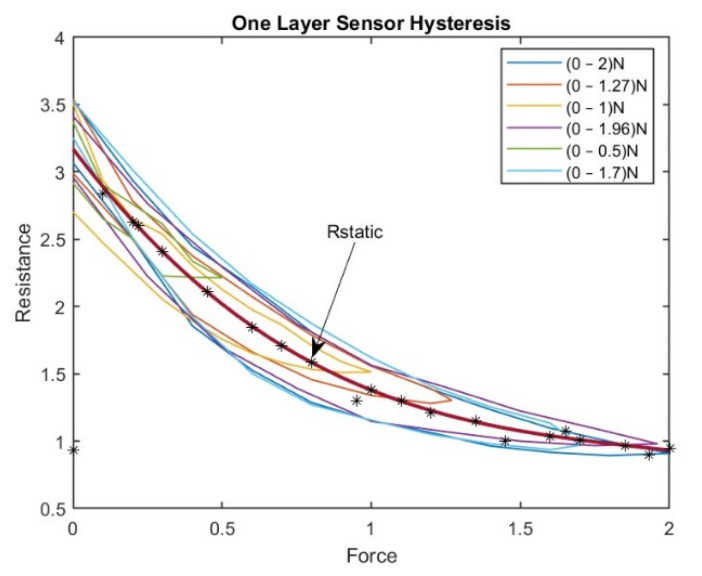
Validation of the system with new experimental results.

It may be highlighted that using the same data set for training and validation can potentially lead to overfitting, where the model becomes highly specialized to the training data and may struggle to generalize to new data. To address this concern, the model was also validated using new and independent data sets. The tactile sensor was tested with new sets of 25, 30, and 60 random forces, and the resulting output resistance was recorded and compared to the expected values (Figure 13). This type of testing ensures that the model can accurately predict resistance values even when presented with new and previously unseen data.

Close alignment of the output results of the BPNN with the target curve R_estimated_ after training is a positive indicator of the effectiveness of the training process. This suggests that the neural network has learned the underlying patterns and relationships between force and resistance, resulting in accurate predictions. This alignment between the predicted results and the target curve indicates that the neural network and, subsequently, the tactile sensor perform well in accurately estimating resistance values.

Continuing testing and validation are essential to ensure the ongoing accuracy and reliability of the tactile sensor. This includes testing the tactile sensor under various conditions and using different data sets that were not included in the training process of the BPNN, to ensure its ability to generalize to new situations and data.

In addition to assessing the performance and accuracy of the BPNN, the evaluation of the robustness of the tactile sensor is also important. Robustness refers to the ability of a sensor to consistently maintain its performance and reliability despite uncertainties or variations in the input.

To evaluate the robustness of the tactile sensor, various factors, including the gradient, mu, and validation check values, were considered for neural network training. Analysis revealed that, at epoch 45, the gradient was 0.0012377, mu was 0.0001, and the validation check value was 6. These values provide useful insights into the stability and adaptability of the neural network during the training process.

**Figure 14:**
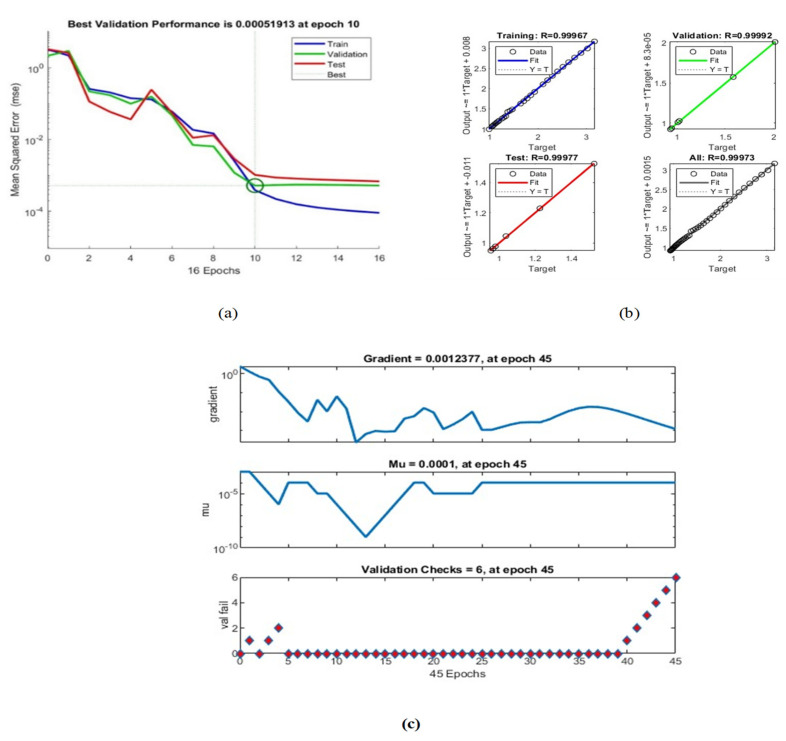
One-Layer Sensor: (**a**) Neural network performance; (**b**) Neural network regression; (**c**) Neural network training.

Furthermore, the validation performance of the BPNN (Figure 14a) indicates the system’s ability to maintain a low validation error of 0.000051913 at a specific epoch. This suggests that the neural network can consistently provide accurate predictions of the tactile sensor’s resistance despite encountering potential variations in the input force. (See Table 2).

Data shown in Figure 14b and the regression values shown in Table 2 reveal that the proposed system exhibits remarkable robustness. The high R-values across all datasets (training, validation, test, and overall) indicate a strong correlation between the predicted resistance values and the actual values. This consistency in performance demonstrates the system’s ability to handle variations in input effectively.

Moreover, utilizing a new dataset for validation, distinct from the one used for training, provides an additional measure of the system’s robustness. By evaluating the system’s performance on unseen data, the generalizability and ability of the system to handle new scenarios can be evaluated. The close alignment between the predicted and the actual results on the validation dataset suggests that the system maintains its accuracy and reliability when faced with new inputs.

Overall, the system’s robustness is the combination of a low validation error, high correlation coefficients, stability during training, and consistent performance on new datasets. These findings highlight the system’s ability to reliably measure changes in resistance based on the applied force, even in the presence of uncertainties or variations in the input. The system’s robustness enhances its practical utility and strengthens its potential for various applications.

By conducting a comprehensive analysis of the system’s performance, accuracy, and robustness, we gain valuable insights into its behavior and capabilities. These insights can assess the sensor system’s design, optimization, and applicability, thereby contributing to improved reliability and performance of the system in real-world scenarios.

Figure 14 shows the validation performance of the BPNN, with the best validation performance of 0.000051913 at a specific epoch. This indicates that the neural network can accurately predict the sensor’s resistance based on the input force applied.

Figure 14b shows the neural network regression values, with R-values indicating the correlation between the predicted and actual resistance values in training, validation, and test datasets. The R-values in training, validation, and testing are all above 0.9999, indicating a strong correlation between the predicted and actual resistance values. The overall R-value of 0.99973 also indicates a strong correlation between predicted and actual values.

These results suggest that the proposed system performs well and accurately predicts the sensor’s resistance. In the expression 1*Target the “*” is used to perform the matrix multiplication between the scalar value 1 and the matrix Target. This operation will multiply each element of the matrix Target by 1.

Table 3 below displays the results of the BPNN evaluation for sensors with varying numbers of layers. The performance of the BPNN was assessed using the mean squared error (MSE) and correlation coefficient (R) metrics.

The results shown above are based on testing different types of sensors with different numbers of layers. The sensors were evaluated using MSE and R.

Mean squared error is a measure of the difference between the predicted values and the actual values. The lower the MSE, the better the performance of the sensor. A correlation coefficient measures the strength and direction of the relationship between predicted and actual values. The closer the correlation coefficient is to 1, the stronger the relationship between the predicted and actual values.

The results show that the performance of the sensors varies depending on the number of layers. Generally, increasing the number of layers in the sensor improves its performance, as shown by the decreasing values of MSE and increasing R values. However, in some cases, the sensor’s performance decreases as the number of layers increases.

These results provide valuable insights for designing and optimizing sensors for various applications.

Results obtained from testing the BPNN for a tactile sensor with varying layers demonstrate that the proposed method is effective in modifying hysteresis nonlinearity in soft tactile sensors based on piezoresistance materials. The BP algorithm adjusted the neural network weights and brought about good convergence and high accuracy in compensating for the effect of hysteresis. The BPNN presented in this paper exhibits promising potential for enhancing tactile sensors by considering the sensor’s range as a variable. To comprehensively assess its versatility, it is recommended to incorporate additional parameters such as the sensor’s geometry size. This enhancement would broaden its applicability across diverse tactile sensing tasks. While the current findings are substantial, the inclusion of supplementary variables in the future holds the potential for even more notable advancements.

The significance of certain sensor designs and materials that have garnered substantial acceptance within the research community is acknowledged, even though the primary focus in this paper centers on the application of the BPNN approach for sensor analysis. Valuable insights and innovative approaches to sensor technology are offered by these studies, as outlined in the provided papers, which complement and enrich the landscape of our current research.

An inkjet-printed tactile sensor with an impressive combination of wide pressure detection range and high sensitivity was introduced by the work of Lee et al. [47]. The utilization of inkjet printing technology and the design of a multi-layered architecture showcase how precise fabrication techniques can lead to enhanced tactile sensing capabilities. Similarly, the potential of a structured, flexible, multi-layered, paper-based pressure sensor for applications in human–machine interfacing was highlighted by the research conducted by Sakhuja et al. [48]. These sensor designs offer a valuable perspective on integrating sensor technology into seamless interactions between humans and machines.

Furthermore, a structural engineering approach to designing flexible capacitive pressure sensors using foil-based materials was presented in the study conducted by Mishra et al. [49]. This strategy capitalizes on the inherent benefits of flexibility and adaptability in sensor design, while also presenting challenges in maintaining a delicate balance between sensitivity and durability. A novel concept of paper-based capacitive touch pads was introduced in the paper authored by Mazzeo et al. [50], showcasing an environmentally friendly and cost-effective alternative for interactive interfaces. Although the immense potential for various industries is held by these designs, potential limitations related to the longevity and stability of paper-based materials need to be considered.

Additionally, the prospects of paper-based piezoresistive MEMS sensors were investigated in the research conducted by Liu et al. [51], aligning with the growing trend of disposable medical devices and wearable electronics. However, this innovative approach, although promising, requires strategies to address challenges associated with maintaining sensor sensitivity and reliability in demanding contexts. Lastly, the impact of diaphragm shape on the performance of foil-based capacitive pressure sensors was delved into by the study carried out by Khan et al. [52]. Insights into the potential of optimizing sensor designs are offered by this study while emphasizing the necessity of overcoming challenges associated with experimental setups and precise characterizations.

By acknowledging the merit and challenges of these diverse sensor designs and materials, a comprehensive understanding of the field’s current state and its potential directions is gained. As this manuscript advances the field through the application of the BPNN approach for sensor analysis, the value of these studies in further enhancing sensor technology and driving innovation across various industries is recognized.

While this paper primarily focuses on presenting the prototype and experimental outcomes, it is important to highlight the real-world potential of these findings. The experiments conducted encompass foundational motions that serve as the basis for various real-world applications, such as reaching for objects and performing bending motions. These initial tests pave the way for considering more complex scenarios in the future.

This study contributes significantly by proposing a methodology to enhance tactile sensor performance by effectively capturing the nonlinear relationship between force and resistance values. This is achieved through a synergistic combination of polynomial regression and neural network techniques. The resultant models offer enhanced interpretability and ease of understanding, compared to the intricate mathematical models such as Preisach or Prandtl–Ishlinskii, rendering them more suitable for practical implementation across a spectrum of fields including robotics, medical devices, consumer electronics, and gaming. However, the selection of the appropriate modeling technique depends on various factors, such as the specific application, the complexity of the hysteresis behavior, and the availability of data.

The BP neural network presented in this paper exhibits promising potential for enhancing tactile sensors by considering the sensor’s range as a variable. To comprehensively assess its versatility, it is recommended to incorporate additional parameters such as the sensor’s geometry size. This enhancement could broaden its applicability across diverse tactile sensing tasks. While the current findings are substantial, the inclusion of supplementary variables in future research holds the potential for even more notable advancements.

In conclusion, the integration of machine-learning techniques showcased in this study significantly enhances the performance of tactile sensors, marking a substantial advancement in current research and paving the way for promising avenues of exploration within this dynamic field. A comprehensive examination of the advantages and challenges inherent in various sensor approaches discussed across the reviewed papers offers profound insights into their potential trajectories and the obstacles they may face in future applications. The research paper by Lee et al. [47] underscores the precision achieved through inkjet printing and multilayered architecture, resulting in heightened sensitivity and a wide pressure detection range. However, the intricacies of calibration and the need for sustained durability present formidable challenges that must be overcome to fully harness this potential.

Similarly, the study by Sakhuja et al. [48] emphasized the benefits of structured design in human-machine interfacing, yet it grapples with the challenge of ensuring long-term reliability and robustness across varying environmental conditions. The work presented by Mishra et al. [49] explores the flexibility of foil-based materials for adaptable applications, but the ongoing challenge lies in striking the delicate balance between sensitivity and durability, while further refining manufacturing processes.

Likewise, the innovation of capacitive touch pads by Mazzeo et al. [50] capitalizes on the eco-friendliness and cost-effectiveness of paper, revolutionizing capacitive touch interfaces. However, the potential limitations of paper material longevity and stability necessitate effective mitigation strategies to ensure sustained accuracy over time. The cost-effective and sustainable solutions in paper-based piezoresistive MEMS sensors, as investigated by Liu et al. [51], align with the trend of disposable medical devices and wearable electronics. Nevertheless, addressing challenges in maintaining sensitivity and reliability in demanding contexts requires innovative approaches.

Lastly, the study conducted by Khan et al. [52] delved into the prospect of optimized designs through meticulous consideration of diaphragm shapes. Although this avenue holds promise, addressing challenges related to rigorous experimental setups and calibration methodologies is vital to ensure precise characterizations. As a result, the acknowledgment and adept navigation of these multifaceted challenges play a pivotal role in unlocking the full potential of these sensor approaches. Overcoming these obstacles will empower researchers to steer the trajectory of sensor technology, ushering in a new era of innovation, reliability, and versatility that is poised to reshape various industries.

## 9. Conclusions

The focus on conductive fiber-based tactile sensors in the current study contributes immensely to the extant knowledge by addressing the distinctive challenges and characteristics associated with this sensor type. By leveraging the capabilities of BPNN, the proposed method modifies the hysteresis nonlinearity, thereby augmenting the accuracy and performance of conductive fiber-based tactile sensors in force measurement and control applications. The proposed method reduced the maximum hysteresis error from 24.2% of the sensor’s full-scale output to 13.5%.

Overall, the paper’s contribution lies in the application of BPNNs to address hysteresis in conductive fiber-based tactile sensors. While using BPNNs for hysteresis approximation is not new, the current study specifically focuses on the challenges and requirements unique to such sensors, pushing the boundaries of tactile sensing and opening up new possibilities for enhancing force measurement and control in relevant applications. In this specific case, the estimated mean distribution for the R-value was found to be 1.1125. This value serves as a measure of central tendency, indicating the average resistance value in the population. Additionally, the standard deviation of 1.513 indicates the spread of or variability in resistance values from the mean.

Overall, tactile sensors are an array of force sensors that provide pressure maps, and many of them are based on polymers due to their ability to conform to different surfaces and withstand high forces at a low cost. However, these sensors often suffer from hysteresis and drift, negatively impacting sensor performance. Various modified Preisach and Prandtl–Ishlinskii (PI) models compensate for these errors and asymmetrical hysteresis nonlinearities in the proposed model. In this research, an attempt was made to improve soft tactile sensors based on piezoresistance materials. These sensors are promising for applications with a large area but suffer from hysteresis, which reduces their accuracy. To address this issue, we developed a BPNN to modify the hysteresis nonlinearity of conductive fiber-based tactile sensors.

In the current study, multiple sensor units were developed with different layers, and the output resistance was collected by applying force sequences on the sensors. These force sequences, along with the corresponding corrected resistance values, were utilized to train a BPNN. This network exhibited good convergence and demonstrated high accuracy during the training process. To evaluate the effectiveness of the trained BPNN, validation experiments were conducted using new datasets. The results of these experiments revealed a significant reduction in the maximum error caused by hysteresis. This reduction signifies the BPNN’s ability to effectively address nonlinearity in soft tactile sensors, resulting in improved accuracy.

The proposed approach outlined in this paper holds immense promise for enhancing the accuracy of soft tactile sensors. Its potential implications span across various applications, including robotics, prosthetics, and human–computer interfaces, where precise and reliable sensing capabilities are of utmost importance. By advancing the accuracy of tactile sensors, this research contributes immensely to the existing knowledge in the field and paves the way for future advancements in practical implementations.

## Figures and Tables

**Table 1:** Summary of Hysteresis Compensation Technique using Backpropagation Neural Network for Soft Sensors.

Approach	Sensor Type	Design/Material	Min Contribution
Adaptation of Control System	Tactile Sensors	N/A	Compensates hysteresis in Robotic System [1]
Recurrent Neural Network	Piezoelectric Actuators	Recurrent Neural Network(RNN)	Models hysteresis for accuracy improvement [2]
Prandtl–Ishlinskii Model	Tactile Sensors	Generalized Prandtl–Ishlinskii Model	Real-time sensor output estimate [3]
Innovative Design	Tactile Sensors	Soft Tactile Electronic Skin	Reduces mechanical/electrical memory effects [4]
Channel-Crack Design	Vibration Sensors	Suspended Sensing Membrane	Enhances sensitivity and stability [5]
Gaussian Process	Tactile Sensors	Gaussian Process with Sensory Markov Properties	Models hysteresis behavior for accuracy [6]
Inverse Feedforward Control	Smart-Material Systems	Preisach Model	Compensates for nonlinearities [7]
Generalized Prandtl–Ishlinskii Model	Micro Positioning Control	Generalized Prandtl–Ishlinskii Model	Improves accuracy in positioning control [8]
Modified PI Model	Piezoelectric Actuators	Modified Prandtl–Ishlinskii Model	Captures asymmetric hysteresis behavior [9]
BP Neural Network Method	Soft Sensors	Backpropagation Neural Network (BPNN)	Mitigates hysteresis nonlinearity [11]
Low-Cost Pressure Sensor	Pressure Sensor Matrix	Flexible Pressure Sensor Matrix	Monitors stroke patients during physiotherapy [13]
Recurrent Neural Network	Soft Sensors	Recurrent Neural Network (RNN)	Mitigates hysteresis in soft sensors [14]
Radial Basis Function NN	Soft Sensors	Radial Basis Function Neural Network	Models and corrects hysteresis in soft sensors [15]
Fuzzy Neural Network	Soft Sensors	Fuzzy Neural Network	Models and compensates hysteresis in soft sensors [16]
Hybrid Neural Network	Soft Sensors	Hybrid Neural Network	Enhances hysteresis compensation in soft sensors [17]
Alternative Sensing Methods	Various Sensors	Capacitive, Piezoelectric, Optical, etc.	Offers alternatives to tackle hysteresis [18]
Silicon Piezoresistive Sensors	Pressure Sensors	Various design principles and considerations	Examines design factors for accuracy and performance [19]
Backpropagation Neural Network (BPNN)	Soft Sensors based on piezoresistive materials	Piezoresistive materials	Modifies hysteresis nonlinearity in soft sensors, enhancing accuracy and overcoming hysteresis limitations. (this work)

**Table 2:** Regression Results and Accuracy Statistics of Datasets.

Dataset	R-Value
Training	0.999967
Validation	0.99992
Test	0.999977
Overall	0.99973

**Table 3:** Comparison of Neural Network Performance with Different Numbers of Layers in the Sensors.

Layers	Observation	MSE	R
1	6	9.41 × 10^−5^	0.996
1	11	0.0039	0.9975
1	14	0.0515	0.9992
1	18	0.0263	0.9988
1	21	0.0058	0.9997
1	29	9.9327 × 10^−5^	0.999
1	46	0.0261	0.9972
3	6	6.7356 × 10^−5^	0.999
3	11	0.0045	0.9983
3	14	0.0515	0.9992
3	18	0.1109	0.9997
3	21	0.0028	1
3	29	0.1510	0.9973
3	46	0.0114	0.9984
6	6	0.0001	1
6	11	0.0055	0.9997
6	14	0.0107	0.9998
6	18	0.1736	0.9995
6	21	0.0051	1
6	29	0.0051	1
6	46	0.0042	0.9994
12	6	0.0034	0.9992
12	11	0.0222	0.9985
12	14	0.0842	0.9989
12	18	0.9985	0.9993
12	21	0.2538	0.9965
12	29	0.9731	0.9728
12	46	0.2597	0.9964

## Data Availability

MDPI Research Data Policies at https://www.mdpi.com/ethics.
